# Commentary on “The Classification of Nerve Injury Revisited: Sundland 0-VI”: Toward a Roadmap for Surgical Decision Making in Nerve Injury

**DOI:** 10.1177/22925503261453810

**Published:** 2026-06-10

**Authors:** Emily M. Krauss

**Affiliations:** 1 Division of Plastic Surgery, Department of Surgery, 12361 Dalhousie University, Halifax, Nova Scotia, Canada

In the article “*The Classification of Nerve Injury Revisited: Sunderland 0-VI*” the authors seek to update the clinical utility of nerve injury classifications and the collective understanding of nerve injuries.^1^ Nerve injury classification is important for surgeons in surgical decision-making: which nerve injuries will improve on their own and which require surgery to optimize recovery. A recent Canadian paper illustrated that surgeons primarily rely on the nerve conduction studies and electromyography that describe an injury to direct surgical decision-making in nerve injury.^2^ Considering the reliance of surgeons on the electrodiagnostic studies to determine injury classification and its potential to recover, this is a timely paper.

Dr. Susan Mackinnon, the senior author, has continued to innovate with respect to our understanding of nerve injury and reconstruction throughout her career, and previously modified the Sunderland classification to include VI, a mixed nerve injury.^3,4^ In this article, she and her co-authors advance the classification to include a new concept of ischemic block with normal electrodiagnostic studies as a Sunderland 0 degree of nerve injury. The concept of a 0-degree nerve injury accounts for nerve compression presentations that previously would have been considered “subclinical” and may be useful when considering diverse compressive nerve injuries such as peroneal neuropathy,^5^ ulnar neuropathy,^6^ and other anecdotal scenarios of sudden improvement after simple decompression. The existence of an ischemic block with normal electrodiagnostic studies was recognized by Sunderland but not included in his original classification, as discussed in this paper, and is reflected in current literature including improved symptoms after cubital tunnel release in a recent series of individuals with ulnar nerve symptoms and negative electrodiagnostic tests.^6^ In this regard, the inclusion of a modified Sunderland classification to include 0-degree injury provides a framework for classification and understanding that until this paper has been talked about in close academic circles but not easily accessible to the practicing surgeon.

This paper is a useful resource for practicing surgeons and essential reading for resident trainees due to its instructive case examples and discussion on the interpretation of electrodiagnostic studies. Perhaps this paper is a step toward a road map in nerve surgery, distilling information often transferred through fellowship training but now presented for everyone. As with her surgical videos, Dr. Mackinnon continues to demystify nerve injury and reconstruction, ensuring a legacy of quality surgical decision-making for nerve injury across the globe and for future surgeons.

## References

[1] Pripotnev S Llaneras NS Teixeira B , et al. The classification of nerve injury revisited: Sunderland 0-VI. Plast Surg. 2025. doi:10.1177/22925503251375862 PMC12550730 41141462

[2] Stephens T Bristol S Chapman KM , et al. Understanding surgical decision-making in patients with traumatic upper extremity peripheral nerve injury: a retrospective cohort study. J Plast Reconstr Aesthet Surg. 2025;104(1):407–413. doi:10.1016/j.bjps.2025.02.022 40174258

[3] Sunderland S . A classification of peripheral nerve injuries producing loss of function. Brain. 1951;74(4):491–516. doi:10.1093/brain/74.4.491 14895767

[4] Mackinnon SE . Nerve surgery*.* Thieme; 2015.

[5] Peters BR Pripotnev S Chi D Mackinnon SE . Complete foot drop with normal electrodiagnostic studies: Sunderland “zero” ischemic conduction block of the common peroneal nerve. Ann Plast Surg. 2022;88(4):425–428. doi:10.1097/SAP.0000000000003053 34864748

[6] Townsend CB Katt BM Tawfik A , et al. Functional outcomes of cubital tunnel release in patients with negative electrodiagnostic studies. Plast Reconstr Surg. 2023;152(1):110e–115e. doi: 10.1097/PRS.0000000000010185 36728488

